# Preretinal hemorrhages following chiropractor neck manipulation

**DOI:** 10.1016/j.ajoc.2018.04.017

**Published:** 2018-04-19

**Authors:** Yannis M. Paulus, Nicholas Belill

**Affiliations:** a Kellogg Eye Center, University of Michigan, Ann Arbor, MI, USA; b Belill Eye Care, Clio, MI, USA

**Keywords:** Chiropractor, Neck manipulation, Preretinal hemorrhage, Complementary and alternative medicine, CAM

## Abstract

**Purpose:**

To report a case of a new complication following complementary and alternative medicine chiropractor neck manipulation with multiple preretinal hemorrhages.

**Observations:**

A 59-year-old Caucasian female presented with the acute, painless constant appearance of 3 spots in her vision immediately after a chiropractor performed cervical spinal manipulation using the high-velocity, low-amplitude technique. Examination multiple unilateral preretinal hemorrhages with no retinal tears. The preretinal hemorrhages were self-limited and resolved by 2 months later.

**Conclusions:**

Chiropractor neck manipulation has previously been reported leading to complications related to the carotid artery and arterial plaques. This presents the first case of multiple unilateral preretinal hemorrhages immediately following chiropractic neck manipulation. This suggests that chiropractor spinal adjustment can not only affect the carotid artery, but also could lead to preretinal hemorrhages.

## 1 Introduction

There is an increasing utilization throughout the world of complementary and alternative medicine (CAM), particularly chiropractic manipulation and massage, to treat a host of medical conditions.^1^ In fact, Americans are estimated to make more visits to CAM practitioners than primary care physicians.^1^ In addition, 72% of those patients using CAM did not report this to their physicians.^1^ With the increasing utilization of CAM, it is critical that we understand the ophthalmic manifestations and complications of CAM use.

Upper spinal manipulation with chiropractors has been associated with a host of ophthalmic events, primarily from effects on the carotid artery and arterial plaques. Stroke,^2^^,^^3^ carotid dissection,^4^ central retinal artery occlusion,^5^^,^^6^ nystagmus,^7^ Wallenberg syndrome,^8^ ophthalmoplegia,^9^ Horner syndrome,^10^ loss of vision,^11^^,^^12^ diplopia,^13^ and ptosis^14^ have all been reported following chiropractic manipulation. We present the first case of unilateral, multiple preretinal hemorrhages immediately following upper spinal chiropractic manipulation.

## 2 Case report

A 59-year-old Caucasian female presented with the acute, painless constant appearance of 3 spots in her vision. She described the spots as “tadpoles” that were constantly present in her vision. She noted the first spot while driving home immediately following a chiropractor neck adjustment, and became more aware that there were 2 additional spots the following day. She received cervical spinal manipulation using the high-velocity, low-amplitude (HVLA) technique on the posterior neck. She also received twisting of the neck that day where it was twisted both to the left and to the right. She had a history of headaches, psoriasis, and restless leg syndrome for which she takes Topamax (topiramate), Stelara (ustekinumab), and Flexeril (cyclobenzaprine), respectively, but denied any further medical history. She denied recent trauma or surgeries.

**Fig. 1:**
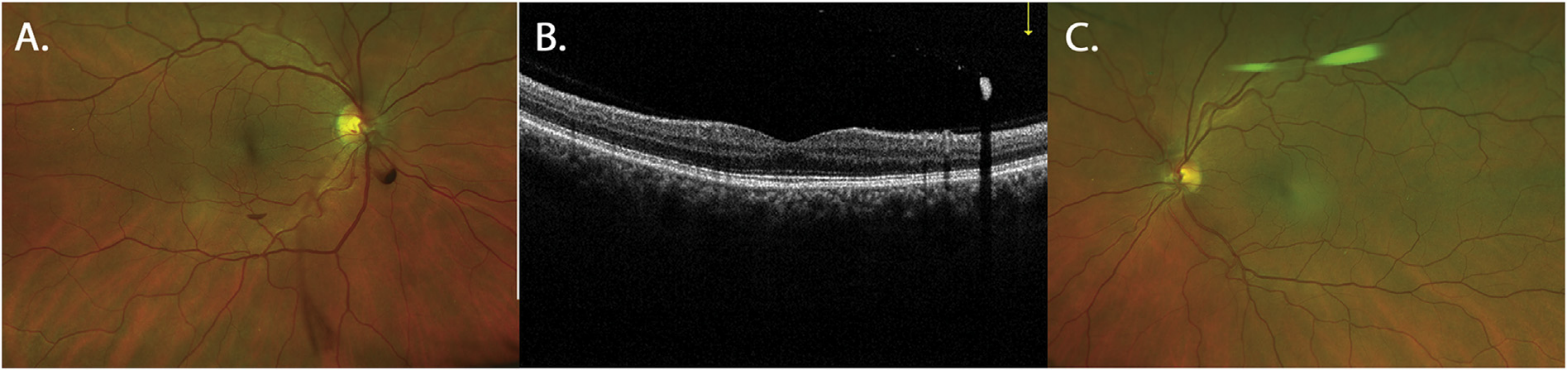
**Imaging on presentation demonstrating preretinal hemorrhages right eye.** A. Optos ultra-widefield scanning laser ophthalmoscope (SLO) image (Daytona, Optos, Dunfernline, Scotland, UK) of the right eye on presentation demonstrating preretinal boat-shaped hemorrhage in the inferior macula, inferonasal to the optic nerve, over the optic nerve head, and inferiorly in the vitreous cavity in a comma shape with no vascular abnormality. B. Optovue spectral domain optical coherence tomography (SD-OCT, Optovue Inc, Fremont, CA, USA) image demonstrating the preretinal hyperreflective hemorrhage on the top right with shadowing behind. C. Optos SLO image of the left eye on presentation demonstrating no hemorrhage or vascular abnormality.

**Fig. 2:**
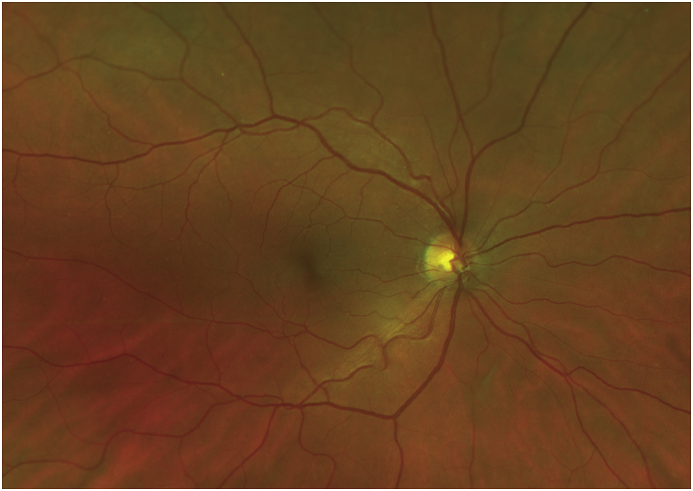
**Imaging 2 months later.** Optos scanning laser ophthalmoscope (SLO) image right eye 2 months after presentation with interval resolution of all preretinal hemorrhage and normal fundus appearance.

Visual acuity was 20/20 OU with minimal refractive error, full extraocular movements, and no ptosis. Neurologic examination was unremarkable. Blood pressure was 123/79, and work-up was negative for diabetes or blood dyscrasia. Slit lamp examination of the right eye demonstrated multiple unilateral preretinal hemorrhages with 3 present inferiorly along with a hemorrhage over the optic nerve (Fig. 1A) and a shallow, incomplete posterior vitreous detachment. Optical Coherence Tomography (OCT) demonstrated the pre-retinal location of the hemorrhage (Fig. 1B). Dilated fundus examination was otherwise unremarkable with normal vasculature including no plaques noted in the vessels, no nonperfusion, and no neovascularization. Scleral depression demonstrated no retinal tears, breaks, or detachments. The left eye was unremarkable (Fig. 1C). The patient's symptoms improved rapidly over 2 weeks. When seen for follow-up 2 months later, the hemorrhages were self-limited and there was resolution of the preretinal hemorrhages with no interval retinal tears (Fig. 2).

## 3 Discussion

Complementary and alternative medicine (CAM) is being increasingly utilized by patients for a host of medical conditions, often without informing physicians.^1^ Chiropractic manipulation has been associated with numerous eye conditions, primarily due to the carotid artery being adjacent to the region of adjustment and manipulation, which in rare cases can lead to carotid dissection. Approximately 100 cases of stroke have been reported following chiropractic manipulation.^2^^,^^3^ Chiropractor adverse effects on the eyes have also been reviewed and are thought to be related to arterial wall dissection,^4^ including central retinal artery occlusion,^5^^,^^6^ nystagmus,^7^ Wallenberg syndrome,^8^ ophthalmoplegia,^9^ Horner syndrome,^10^ loss of vision,^11^ hemianopsia (likely from stroke),^12^ diplopia,^13^ and ptosis.^14^

Upper spinal manipulation with the HVLA technique involves high velocity, low-amplitude thrusts on the cervical spine administered posteriorly. No other etiology of the preretinal hemorrhages was found on work-up (no leukemic retinopathy, hypertension, diabetes, or retinal tear). The temporal association immediately while driving home from the chiropractic procedure makes other causes less likely, although we cannot exclude Valsalva retinopathy or progressive posterior vitreous detachment. Given the lack of any retinal vessel abnormalities or plaques along with the temporal association, we postulate that the chiropractor neck manipulation itself induced vitreo-retinal traction that likely led to preretinal hemorrhages which were self-limited. It is also possible that the HVLA technique could have mechanically assisted with induction of a posterior vitreous detachment.

The ophthalmic manifestations of chiropractor neck manipulation has primarily been viewed previously from its effect on the carotid artery and subsequent central retinal artery along with adjacent neurons, but this case demonstrates that it can also lead to multiple preretinal hemorrhages.

## 4 Conclusions

Chiropractor neck manipulation has previously been reported leading to complications related to the carotid artery (eg, dissection) and arterial plaques. This presents the first case of multiple acute, unilateral preretinal hemorrhages immediately following cervical spinal manipulation using the high-velocity, low-amplitude technique on the posterior spine that spontaneously resolved.

## Patient consent

The patient consented to the submission and publication of this case report with documentation on file.

## Acknowledgements and disclosures

### Funding

Funding was provided in part by the National Eye Institute Michigan Vision Clinician-Scientist Development Program 4K12EY022299 (YMP).

### Conflicts of interest

None of the authors has a conflict of interest with the submission.

### Authorship

All authors attest that they meet the current ICMJE criteria for authorship.
